# Mimicking the Dystrophic Cardiac Extracellular Environment through DystroGel

**DOI:** 10.1002/adhm.202404251

**Published:** 2025-02-17

**Authors:** Maila Chirivì, Fabio Maiullari, Marika Milan, Maria Grazia Ceraolo, Nicole Fratini, Alessandra Fasciani, Salma Bousselmi, Michael Stirm, Francesca Scalera, Francesca Gervaso, Michela Villa, Raffaello Viganò, Francesca Brambilla, Pierluigi Mauri, Elena De Falco, Dario Di Silvestre, Marco Costantini, Eckhard Wolf, Claudia Bearzi, Roberto Rizzi

**Affiliations:** ^1^ Department of Molecular Medicine Sapienza University Viale Regina Elena, 324 Rome 00161 Italy; ^2^ Neurology Unit Fondazione IRCCS Ca' Granda Ospedale Maggiore Policlinico Via Francesco Sforza, 35 Milan 20122 Italy; ^3^ Ph.D. Program in Cellular and Molecular Biology Department of Biology University of Rome “Tor Vergata” Via della Ricerca Scientifica, 1 Rome 00133 Italy; ^4^ Fondazione Istituto Nazionale di Genetica Molecolare “Romeo ed Enrica Invernizzi” Via Francesco Sforza, 35 Milan 20122 Italy; ^5^ Chair for Molecular Animal Breeding and Biotechnology Gene Center and Department of Veterinary Sciences LMU Munich 81377 Munich Germany; ^6^ Center for Innovative Medical Models (CiMM) Department of Veterinary Sciences LMU Munich 85764 Oberschleißheim Germany; ^7^ Institute of Nanotechnology National Research Council c/o Campus Ecotekne via Monteroni Lecce 73100 Italy; ^8^ Department of Biosciences University of Milan Via Celoria, 26 Milan 20133 Italy; ^9^ Institute for Biomedical Technologies National Research Council Via Fratelli Cervi, 93, Segrate Milan 20054 Italy; ^10^ Department of Medical‐Surgical Sciences and Biotechnologies Sapienza University of Rome C.so della Repubblica 79 Latina 04100 Italy; ^11^ Institute of Physical Chemistry – Polish Academy of Sciences Marcina Kasprzaka 44/52 Warsaw 01–224 Poland

**Keywords:** biomaterials, duchenne muscular dystrophy, extracellular matrices, heart failure, muscular dystrophy, pathological hydrogel

## Abstract

Advances in understanding the mechanisms behind genetic diseases like Duchenne muscular dystrophy (DMD) underscore the critical role of the extracellular matrix (ECM) composition in disease progression. Effective in vitro models must replicate the intercellular relationships and physicochemical properties of native ECM to fully capture disease‐specific characteristics. Although recent biomaterials support the in vitro biofabrication of pathophysiological environments, they often lack disease‐specific ECM features. In this study, DystroGel, a hydrogel derived from the cardiac ECM of a porcine DMD model, replicates the distinct molecular composition of dystrophic cardiac tissue for the first time. The findings indicate that the dystrophic ECM matrix exhibits a unique protein profile, impacting cellular processes critical to DMD pathology. This work demonstrates the importance of using a 3D substrate that recreates intercellular dynamics within a defined pathological environment, enhancing the ability to model genetic disorders and providing a valuable tool for advancing personalized therapeutic strategies.

## Graphical abstract

This research introduces DystroGel, a cutting‐edge biocompatible pathological hydrogel derived from decellularized dystrophic porcine heart tissue, suitable for tissue modeling applications. Rheological and proteomic analyses reveal structural and protein composition modifications, underscoring its unique properties. Notably, DystroGel influences gene expression of encapsulated fibroblasts, cardiomyocytes, and neurons, highlighting its bioactive biological potential to mimic the dystrophic ECM.

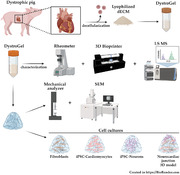

## 1 Introduction

In DMD, extensive remodeling affects multiple muscle groups, notably the heart, disrupting intercellular communication and the synchrony of myocardial contractions. This disruption in cellular synchrony significantly reduces cardiac efficiency and frequently progresses to heart failure (HF). HF in DMD patients, largely due to dilated cardiomyopathy, presents a significant therapeutic challenge. With advances in respiratory and other supportive therapies, cardiovascular disease has become the leading cause of death in DMD patients.^[^ ^1^ ^]^ By age 30, nearly all DMD patients experience heart failure, and despite the effectiveness of standard treatments, HF is responsible for over 50% of deaths in this population.^[^ ^2^, ^3^ ^]^ The abnormal physiochemical remodeling of the ECM plays a central role in these complications, disrupting critical signaling pathways and altering cellular architecture across disorders, including muscular dystrophy.^[^ ^4^, ^5^ ^]^ The ECM contains numerous proteoglycans, primarily from the small leucine‐rich proteoglycan family, including glycosaminoglycans like chondroitin and dermatan sulfates.^[^ ^6^ ^]^ It also contains key structural proteins such as collagens, elastin, fibronectin (FN), and laminins.^[^ ^7^, ^8^, ^9^ ^]^ Proteoglycans bind to collagen at specific sites and are sensitive to changes within the ECM; when these interactions become unbalanced, cellular dysfunction can occur, potentially leading to cell death.^[^ ^10^ ^]^ The growing understanding of ECM's role in maintaining cell communication and tissue homeostasis emphasizes the need to replicate these dynamics in vitro, especially to study pathological conditions like DMD. Currently, many research groups are shifting toward the in vitro recapitulation of complex hetero‐cellular systems to understand the molecular dynamics occurring during organogenesis in both physiological and pathological contexts. DMD and other dystrophies, characterized by progressive muscle degeneration, present complex challenges that require a deep understanding of tissue mechanics.^[^ ^11^, ^12^ ^]^ By examining how dystrophic tissues endure mechanical stress, we can elucidate fundamental mechanisms, including muscle degeneration, fibrosis, and diminished contractility, thus facilitating novel interventions to decelerate disease progression. Additionally, investigating tissue viscoelasticity yields critical insights essential for developing assistive devices and rehabilitation strategies tailored to the unique biomechanical needs of individuals with dystrophies. Altogether, integrating knowledge of the mechanical and biochemical interactions between cells and ECM not only deepens our understanding of these conditions but also establishes a comprehensive framework for improving functionality, patient care, and quality of life.^[^ ^13^ ^]^

Our hypothesis suggests that the extensive remodeling of dystrophic cardiac ECM is a critical factor in the decline of cardiac performance. In the myocardium of DMD patients, significant alterations in the cytokine profile^[^ ^14^ ^]^ signal pathological ECM remodeling. By investigating the responses of cardiac cell populations to various matrix compositions, we can gain insights into the specific impacts of pathological ECM on myocardial function. Studies have repeatedly demonstrated that surrounding matrices exert synergistic effects on cardiomyocyte maturation,^[^ ^15^ ^]^ highlighting the promise of 3D techniques in constructing cardiac models. Such models could prove invaluable for testing personalized therapies,^[^ ^16^ ^]^ potentially improving both reproducibility and clinical efficacy. To date, numerous biomaterials have been used in efforts to achieve efficient 3D biofabrication capable of replicating the complex pathological conditions induced by the ECM. However, these approaches face inherent limitations.^[^ ^17^, ^18^, ^19^, ^20^, ^21^ ^]^ While several studies have advanced hybrid inks that combine ECM components with natural biomaterials, such as a bioink incorporating methacrylated collagen, laminin‐111, and fibronectin with human induced pluripotent stem cells (iPSCs),^[^ ^22^, ^23^ ^]^ or blends of Gelatin methacrylate (GelMA) and methacrylated hyaluronic acid (Me‐HA) hydrogels with cardiac decellularized ECM (dECM),^[^ ^24^, ^25^ ^]^ their clinical translation remains limited due to challenges in achieving consistent rheological and mechanical properties.

In this study, we present a breakthrough in 3D biofabrication with the development of a biocompatible hydrogel, DystroGel, formulated from cardiac dECM specifically derived from dystrophic pigs. Using a model that exhibits accelerated dystrophic progression,^[^ ^26^ ^]^ we sourced cardiac dECM from pigs at 1‐day and 4‐month‐old to capture the progression of ECM remodeling throughout disease pathology. This novel hydrogel formulation not only replicates the precise molecular composition but also embodies the unique physical properties characteristic of dystrophic cardiac ECM.^[^ ^27^ ^]^ To evaluate DystroGel's biocompatibility and functional properties, we performed extensive testing with fibroblasts and iPSC‐derived cardiomyocytes (iPSC‐CMs), demonstrating its robust biocompatibility. Furthermore, we developed iPSC‐derived sympathetic neurons (iPSC‐SNs) to construct a 3D neurocardiac junction, effectively simulating the sympathetic innervation typically compromised in DMD patients.^[^ ^28^ ^]^ This methodology, leveraging disease‐specific ECM from dystrophic cardiac tissue, represents a significant advancement over traditional biomaterials. By providing an authentic model that precisely mirrors DMD cardiac pathology, DystroGel holds substantial promise for improving the reproducibility and applicability of ECM‐based 3D techniques. Additionally, this approach may facilitate deeper insights into pathologies marked by molecular imbalances, advancing our understanding of impaired intercellular and interorgan communication, irrespective of their underlying causes.

## 2 Results

### 2.1 dECM‐Based Hydrogel Production and Characterization

**Figure 1:**
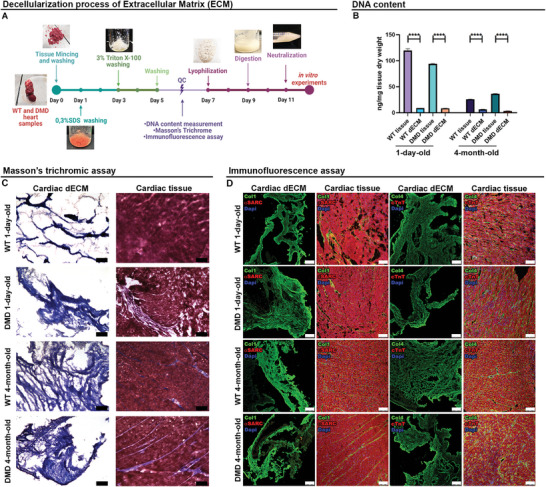
Decellularization process assessment. A) ECM decellularization protocol timeline. B) Quantitative analysis of DNA content. Error bars represent ± SEM. Student's *t‐*test was used to evaluate the differences between means; *n* = 3; significant differences: *p < 0.05, ***p < 0.001, ****p < 0.0001. C) Masson's trichrome assay of 1‐day and 4‐month‐old WT and DMD porcine cardiac native tissue and dECM. Scale bars represent 100 µm. D) Cardiac tissue and dECM of WT and DMD pig stained against Collagen I (green) and aSARC (red), and Collagen IV and cardiac troponin T (cTnT; red). Nuclei were detected by Dapi (blue). The scale bar represents 100 µm.

Pig heart samples from 1‐day (total heart) and 4‐month‐old (right and left ventricles and atria) WT (wild type) and DMD animals were used to refine the ECM decellularization protocol (**Figure** **1**A) for producing a biocompatible biomaterial for subsequent cell encapsulation, based on the protocol described by Pati, et al.^[^ ^29^ ^]^ Cardiac samples, after being smashed into 1 mm thick pieces, were washed with 0.3% SDS for 48 h and then 3% Triton X‐100 for a further 48 h, to eliminate muscle and cellular components. The results demonstrated a significant reduction in DNA content in both WT and DMD cardiac dECM compared to native porcine heart samples (*p* < 0.0001), confirming the successful removal of all cellular components (Figure 1B). Additional validation of the decellularization process was conducted through Masson's trichrome staining (Figure 1C) and immunofluorescence assays targeting collagen I and IV, Anti‐Sarcomeric Alpha Actinin (αSARC), and cardiac troponin T (cTnT) to detect any remaining CMs (Figure 1D). The analyses confirmed the absence of muscle components and nuclei in the decellularized tissues; while both decellularized and native tissues showed significant amounts of collagen I and IV fibrils, suggesting the preservation of crucial elements of the ECM.

**Figure 2:**
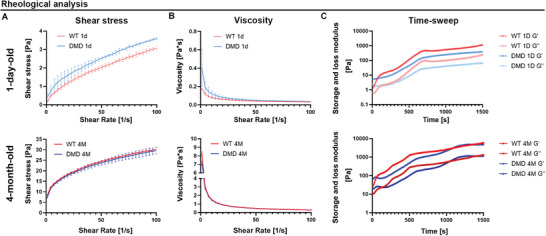
dECM characterization. A) Shear stress, B) viscosity, and C) time‐sweep values of dECM derived from WT and DMD heart tissues of 1‐day‐old (upper panels) and 4‐month‐old (lower panels) pigs.

After quality assessment, decellularized tissues were subjected to lyophilization, digestion, and neutralization in acetic acid and pepsin (10 mg of pepsin per 100mg of lyophilized dECM in 0.5 m acid acetic solution) to produce a thermosensitive hydrogel biomaterial. Furthermore, the rheological properties of the dECM pre‐solutions were investigated by shear rate and time sweep tests at 37 °C (**Figure** **2**). These tests revealed that all dECM pre‐solutions were quite viscous and exhibited specific shear‐thinning behavior within the studied range of shear rates (Figure 2A,B). Notably, dECM pre‐solutions derived from 4‐month‐old pigs showed significantly higher viscosity than those from 1‐day‐old pigs (Figure 2B). In addition, DMD samples from 1‐day‐old pigs showed higher viscosities at low shear rates than WT samples of the same age, indicating differences in composition between the two tissues from birth. Time‐sweep tests were conducted to evaluate the sol–gel transition of the dECM pre‐solutions, observing the evolution of the G′ and G″ traces at the physiological temperature. The results demonstrated that all tested samples were temperature sensitive when held at 37 °C (Figure 2C), with G′ values consistently higher than G″. No crossing of the G′ and G″ traces was observed, indicating rapid gelation of the dECM‐derived hydrogels under physiological conditions.

Finally, pre‐solutions of dECM were mechanically tested. Samples were poured into polydimethylsiloxane (PDMS) molds, cross‐linked at 37 °C, and used to conduct compression tests, in vitro stability tests, and acquire scanning electron microscope (SEM) images.

**Figure 3:**
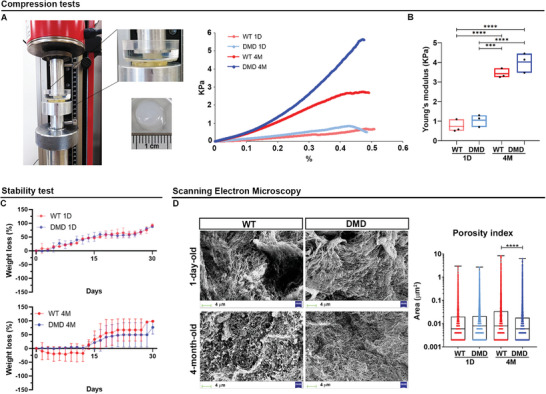
dECM‐based hydrogel characterization. A) Compression tests and B) Young's modulus evaluation of 3D bulks. Error bars represent ± SEM. The repeated measure ANOVA with Tukey correction was used to evaluate the differences between means; *n* = 3; significant differences: *p < 0.05, ***p < 0.001, ****p < 0.0001. C) Weight loss percentage reported in a time frame of 30 days of 1‐day (upper panel) and 4‐month‐old (lower panel) matrices bulks. D) SEM images of WT and DMD dECM 3D bulks and related graphs showing the porosity index. The scale bars represent 2 mm. Error bars represent ± SEM. Student's *t‐*test was used to evaluate the differences between means; *n* = 3; significant differences: *p < 0.05, ***p < 0.001, ****p < 0.0001.

Compression tests revealed that, as the strain increased, the stiffness also increased for all samples, exhibiting the typical compressive stress–strain behavior of biological materials (**Figure** **3**A). Young's modulus values for the dECM derived from 1‐day‐old WT and DMD pig cardiac tissues were 0.73 ± 0.32 and 0.92 ± 0.31 kPa, respectively. While the dECM values of 4‐month‐old WT and DMD pig cardiac tissues were 3.48 ± 0.32 and 4.02 ± 0.50, respectively. dECM derived from 1‐day‐old pig tissues showed very low compressive stiffness for both WT and DMD samples while the average Young's modulus of DMD was slightly higher than that of WT, although not statistically significant. In contrast, dECM derived from 4‐month‐old pig tissues showed significantly higher stiffness than 1‐day‐old tissues (*p* < 0.001). Furthermore, Young's modulus of DMD was higher than that of WT, reflecting the distinct biophysical composition of pathological tissues (Figure 3B). Weight loss rates were calculated to evaluate the in vitro stability of the dECM constructs by exposing the matrices to 37 °C for 30 days. The 1‐day‐old samples showed similar behavior, with a linear degree of degradation and no statistically significant differences (Figure 3C, upper panel).^[^ ^30^ ^]^ In contrast, 4‐month‐old matrices showed a similar trend up to 13 days, after which dystrophic samples displayed a lower degradation rate than WT samples, indicating greater stability (Figure 3C, lower panel).

Successively, the porosity of the dECMs was evaluated by SEM acquisitions. Cross‐sectional surface SEM images qualitatively demonstrated that both 1‐day‐old WT and DMD samples had comparable microstructures, while 4‐month‐old arrays possessed a randomly oriented fibrillar structure with variable pore sizes (Figure 3D). Indeed, image analysis confirmed that DMD samples at 4 months had a porosity index significantly reduced compared to WT samples (*p* < 0.0001). Overall, these results suggest that the 4‐month‐old DMD dECM‐based hydrogel mimics the characteristics of dystrophic cardiac tissue, exhibiting increased stiffness typical of fibrotic tissue, as well as enhanced firmness that prevents disintegration.

### 2.2 Proteomic Analysis

**Figure 4:**
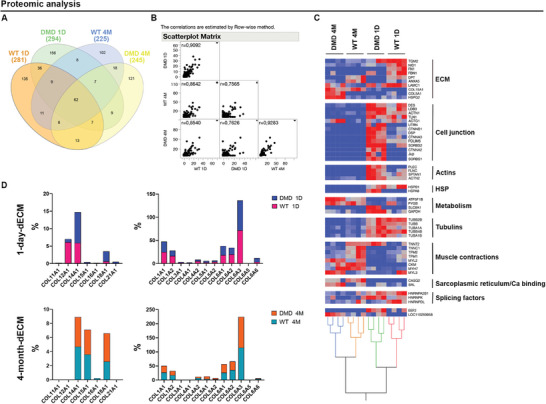
Proteomic analysis. A) Venn diagram of protein identified in 1‐day and 4‐month‐old dECM samples. B) Spearman's correlation of the total protein profiles. C) Hierarchical clustering from DEPs LDA, *p *< 0.05) found by comparing the protein profiles characterized for the investigated phenotypes (*n* = 4 per condition); heat map (normalized SpC) indicates in red and in blue light proteins up‐ and down‐regulated, respectively. D) Different expressions of collagen proteins (%) in 1‐day (upper panel) and 4‐month‐old (lower panel) dECM.

To thoroughly characterize this dECM‐based hydrogel, we employed a holistic proteomic approach using high‐resolution mass spectrometry to profile the proteome and identify differentially expressed proteins (DEPs) in 1‐day and 4‐month‐old WT and DMD dECM samples (**Figure** **4**). This analysis provided a comprehensive overview of ECM protein composition and alterations primarily related to disease progression and aging. On average, we detected over 200 proteins (up to 300) across all phenotypes examined, with ≈100–150 proteins identified specifically per condition (Figure 4A). Notably, 1‐day (mean *r* = 0.9092) and 4‐month‐old (mean *r* = 0.9293) WT and DMD dECM samples showed a strong overall correlation in their total protein profiles when statistically evaluated. In contrast, the lower correlation value between 1‐day and 4‐month‐old DMD dECM samples indicates more substantial proteome remodeling between these conditions (Figure 4B). To provide a systems perspective on the protein changes that occur, we constructed a protein–protein interaction (PPI) network by integrating differentially expressed proteins (*p* < 0.05) with the Sus scrofa interactome (Figure 4C). In 4‐month‐old WT and DMD samples, we observed downregulation of functional modules associated with cell junctions, actins, heat shock proteins (HSPs), tubulins, and splicing factors compared to their 1‐day‐old counterparts, suggesting dysregulation of these signaling pathways in the context of aging. Furthermore, we noted dysregulated expression of proteins involved in muscle contraction. Specifically, myosin light chain 2 (MYL2), M‐type creatine kinase (CKM), myosin‐7 (MYH7), and myosin light chain 3 (MYL3) were upregulated in 4‐month‐old samples compared to younger, while troponin T2 (TNNT2), troponin C (TNNC1), beta‐tropomyosin (TPM2), and tropomyosin alpha‐1 chain (TPM1) proteins were downregulated in 4‐month‐old DMD samples compared to WT. Our investigation uncovered specific enrichment of ECM pathway‐associated proteins in 4‐month‐old DMD dECM, including collagen type V alpha chain 1 (COL5A1) and heparan sulfate proteoglycan 2 (HSPG2). Yokota et al. have previously established a significant correlation between the expression level of COL5A1 and scar size after myocardial infarction, indicating its mechanical role in regulating both cardiac scar size and tissue mechanical properties.^[^ ^31^ ^]^ Furthermore, HSPG2 within the ECM is critical for maintaining tissue structure and function, regulating cellular behavior, and supporting various physiological processes. However, dysregulation of HSPG2 and its interactions within the ECM in specific pathological contexts, such as DMD^[^ ^32^ ^]^ and cancer,^[^ ^33^ ^]^ may herald adverse remodeling and contribute to disease progression. Next, we examined the different compositions of collagens, given their importance as primary constituents of the ECM. Our analysis revealed that the predominant collagens present in the examined matrices were collagen type XIV alpha 1 chain (COL14A1), collagen type XV alpha 1 chain (COL15A1), collagen type XVIII alpha 1 chain (COL18A1) and the A1 and A2 isoforms of type I collagen (COL1A1, COL1A2), as well as A1, A2 and A3 of type VI collagen (COL6A1, COL6A2 and COL6A3) (Figure 4D). Overall, these findings enhance our understanding of the proteomic landscape of this novel pathological dECM‐based hydrogel and its potential applications in tissue engineering and regenerative medicine.

Moreover, since the data obtained from rheological characterization, mechanical stress analysis, and proteomics revealed that the 4‐month‐old DMD dECM‐based hydrogel more closely recapitulates the native matrix characteristics of a dystrophic heart, we selected this time point for performing in vitro experiments. From this point onward, the 4‐month‐old DMD dECM‐based hydrogel will be referred to as DystroGel, highlighting its distinctive properties as a biomimetic matrix.

### 2.3 DystroGel Printability Test

**Figure 5:**
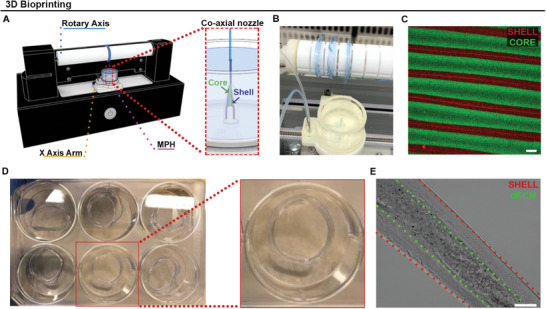
3D Bioprinting test. A) Schematic representation of the 3D bioprinting rotary wet‐spinning (RoWS) platform and the relative magnification of the co‐axial microfluidic printing extruder used to manufacture core/shell hydrogel fibers. B) Representative images of the bioprinting process. C) Fluorescent image of highly aligned fiber bundles characterized by a core/shell architecture. D,E) Representative images of the bioprinted dECM and brightfield image of core/shell fiber. The scale bars represent 100 µm.

3D printing experiments were conducted to validate the printability efficacy and structural integrity of the DystroGel. An innovative biofabrication platform (RoWS bioprinter), inspired by the wet spinning technique, was utilized to create highly anisotropic, engineered myo‐substitutes.^[^ ^34^ ^]^ The platform primarily comprises three components: a microfluidic print head (MPH) for the extrusion of fibers, a rotating drum for fiber collection, and an *x‐*axis arm to control the deposition of 3D samples along the drum (**Figure** **5**A). The MPH includes two inlets, one for the core and one for the shell, connected to the two compartments of the coaxial nozzle (Figure 5B), where the two bioinks merge to form the core/shell fiber (Figure 5C). The fiber is extruded into a cross‐linking bath, facilitating ionic cross‐linking of the alginate chains in the shell phase through calcium ions (Figure 5B). With the alginate‐based shell confinement, this setup enables the bioprinting of a diverse range of bioink cores, including dECM (Figure 5D,E).

Furthermore, the confinement of the core from the external environment greatly extends the time available for crosslinking, making it compatible with slow crosslinking processes such as the thermal crosslinking of dECM. The tested core–shell system supports the possibility of generating 3D dECM‐based constructs without combining dECM with other support biomaterials (GelMA, Alginate, Chitosan),^[^ ^35^, ^36^, ^37^ ^]^ which possess different physicochemical properties and could potentially alter DystroGel characteristics. The printability test conducted on DystroGel revealed its significant potential as a structured bioink material, prompting further evaluation even in the absence of cellular components. Assessing its use in forming intricate architectures is essential before cellular incorporation, as DystroGel is specifically engineered to replicate the cardiopathological environment with a precision that cannot be achieved through conventional methods. By establishing DystroGel's capacity to support structured formations that closely emulate the complex microarchitecture of tissue, we lay the groundwork for a more accurate and functional disease model, ultimately enhancing the relevance and fidelity of this biomimetic scaffold.

This test serves as a proof of concept to illustrate the feasibility of printing our DMD dECM‐based hydrogel, with the understanding that more extensive studies are needed to explore and optimize its application for true bioprinting contexts.

### 2.4 DystroGel Biocompatibility Assay

**Figure 6:**
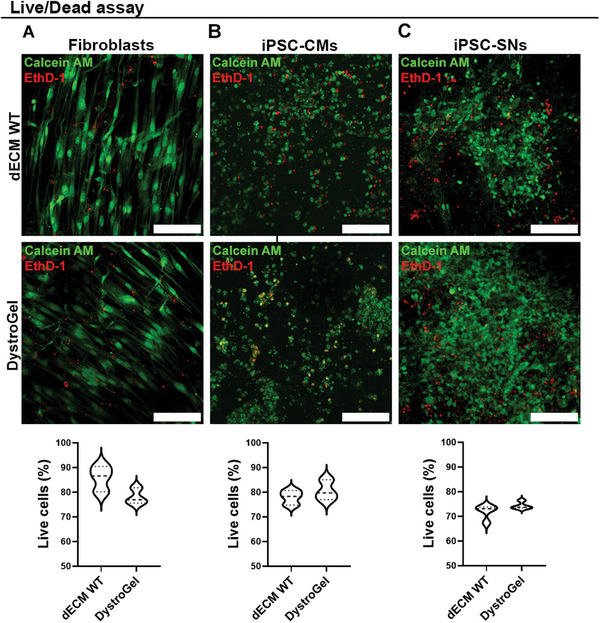
Viability assessment. Representative confocal images and relative graphs of the live and dead assay on A) fibroblasts B) iPSC‐CMs C) iPSC‐SNs cultured for 15 days. The scale bars represent 100 µm. Student *t‐*test was used to evaluate the differences between means; *n *= 3 sections per *n* = 3 biological replicates; significant differences: *p < 0.05, **p < 0.01, ***p < 0.001.

After the characterization, we employed DystroGel to perform experiments simulating cellular conditioning within a dystrophic cardiac environment. Our goal was to establish a more biologically relevant model, thus improving the robustness and significance of our findings. Our hypothesis postulates that the substantial mechano‐biological distinctions observed in previous assays are sufficient to influence cellular behavior and interactions. Therefore, we encapsulated iPSC‐CMsand iPSC‐SNs and human fibroblasts within 3D constructs using DystroGel and healthy matrix, as control. Constructs were cultured for 15 days, after which we assessed the biocompatibility of each cell population within both biomaterials. Analysis of live and dead cells indicated comparable viability between fibroblasts (**Figure** **6**A), iPSC‐CMs (Figure 6B), and iPSC‐SNs (Figure 6C) after 15 days of culture in both matrices, suggesting good biocompatibility of the biomaterial.

**Figure 7:**
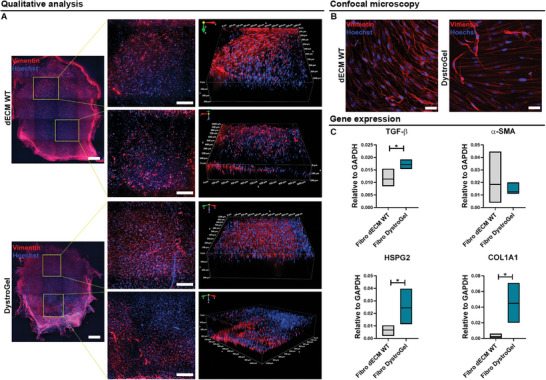
Fibroblasts encapsulation in dECM WT and DystroGel. A) Z‐stack images’ reconstruction of the whole WT (upper panel) and DystroGel (lower panel) samples and higher magnification views of two distinct regions of the specimen. The scale bars represent 500 and 200 µm, respectively. Volumetric 3D reconstruction of the samples at higher magnifications. Fibroblasts were stained with Vimentin (red), and the nuclei were detected with Hoechst (blue). B) Representative confocal images of fibroblasts in dECM WT and DystroGel. The cells were stained against Vimentin (red). Nuclei were counterstained with Hoechst (blue). Scale bars represent 50 µm; C) Quantitative RT‐PCR for the expression of TGF‐b, a‐SMA, HSPG2, and COL1A1 genes in fibroblasts (Fibro). Student *t*‐test was used to evaluate the differences between means; n = 3; significant differences: *p < 0.05, **p < 0.01, ***p < 0.001.

Spinning disk confocal imaging was used to examine the morphology and spreading behavior of fibroblasts within the masses (**Figure** **7**A). Representative 3D image reconstructions illustrated that fibroblasts were evenly distributed throughout the hydrogel, with comparable cell density observed near the surface and center of the masses (Figure 7A). Furthermore, the ability of fibroblasts to spread within the hydrogels was assessed using confocal laser scanning microscopy (Figure 7B). Subsequently, the molecular alterations induced by the biophysical characteristics of the 3D environment were explored through quantitative RT‐PCR, measuring the expression levels of genes associated with fibrosis and ECM remodeling. Specifically, the expression of TGF‐β (dECM DMD vs dECM WT, *p* = 0.0170), HSPG2 (dECM DMD vs dECM WT, *p* = 0.0302), and COL1A1 (dECM DMD vs dECM WT, *p* = 0.0122) was significantly upregulated in fibroblasts encapsulated within the DMD dECM compared to the healthy matrix (Figure 7C). These results suggest that the mechanical tension imposed by the DMD matrix is adequate to induce the expression of TGF‐β, a fundamental mediator in the conversion of fibroblasts into myofibroblasts, thus promoting the activation of matrix synthesis mechanisms.^[^ ^38^, ^39^, ^40^, ^41^ ^]^ However, the expression of α‐smooth muscle actin (α‐SMA), a downstream marker of TGF‐β in the process of epithelial‐mesenchymal transition associated with fibrosis deposition, remained unchanged, indicating that a period of culture more extensive may be necessary for its overall modulation. Additionally, it is well known that increased fibroblast spreading and collagen type I expression occur in stiffer matrices, which may mimic the late stage of myocardial infarction‐related remodeling with scar formation.^[^ ^42^ ^]^

**Figure 8:**
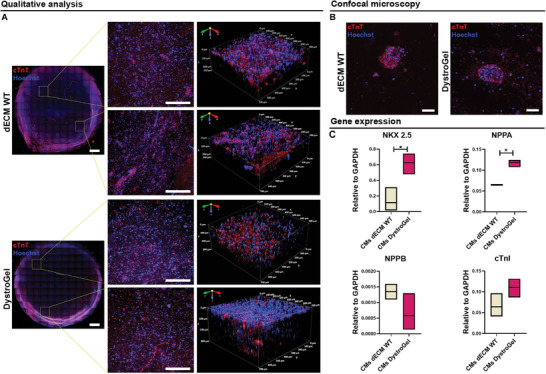
iPSC‐CMs embedded in dECM WT and DystroGel. A) Z‐stack image reconstruction of the whole WT (upper panel) and DystroGel (lower panel) samples and higher magnification views of two distinct regions of the specimen. The scale bars represent 500 and 200 µm, respectively. Volumetric 3D reconstruction of the samples at higher magnifications. The cells were colored with cardiac troponin T antibody (cTnT, red) and the nuclei were detected by Hoechst (blue). B) Representative confocal images of iPSC‐CMs in dECM WT and DystroGel. iPSC‐CMs were colored with cTnT (red). Hoechst (blue) was used to stain nuclei. The scale bars represent 50 µm. C) Relative gene expression related to cardiac early (NKX2.5, NPPA, and NPPB) and late gene (cTnI). Student *t*‐test was used to evaluate the differences between means; *n* = 3; significant differences: *p < 0.05, **p < 0.01, ***p < 0.001.

Similarly, iPSC‐CMs exhibited consistent distribution in both matrices, as visually demonstrated by the 3D volumetric reconstruction images (**Figure** **8**A). Confocal imaging further confirmed the sustained presence of cardiac troponin T (cTnT) post‐encapsulation in the matrices (Figure 7B). Thereafter, the alteration in gene expression induced by different dECMs, both healthy and pathological, was investigated. Specifically, quantitative RT‐PCR was employed to analyze the expression of pertinent early (NKX 2.5, NPPA, and NPPB) and late (cTnI) cardiac genes. Notably, CMs encapsulated in DystroGel exhibited significant upregulation in NKX 2.5 and NPPA gene expression compared to the WT matrix. This intriguing discovery suggests that our pathological dECM may play a significant role in disrupting the maturation of these cells, thereby potentially prompting the reactivation of embryonic pathways—a phenomenon associated with cardiac pathophysiological stress.^[^ ^43^ ^]^

**Figure 9:**
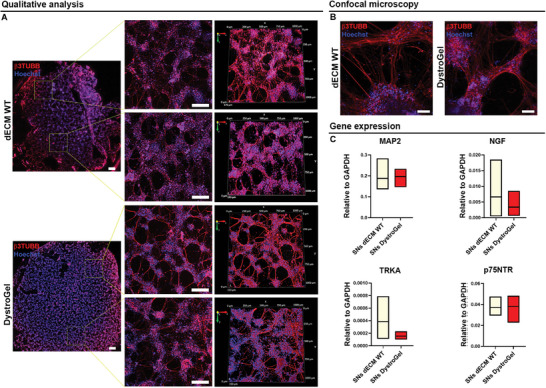
iPSC‐SNs embedded in dECM WT and DystroGel. A) Z‐stack image reconstruction of the whole WT (upper panel) and DystroGel (lower panel) samples and higher magnification views of two distinct regions of the specimen. Scale bars represent 500 and 200 µm, respectively. Volumetric 3D reconstruction of the samples’ higher magnifications. b3TUBB antibody was used to stain iPSC‐SNs (red). Nuclei were counterstained with Hoechst (blue). B) Representative confocal images of iPSC‐SNs in dECM WT and DystroGel. SNs were stained with b3TUBB (red). Hoechst (blue) was used to stain nuclei. Scale bars represent 50 µm. C) Gene expression analysis related to SNs differentiation (MAP2) and survival (NGF, TRKA, and p75NTR). Student *t*‐test was used to evaluate the differences between means; *n* = 3; significant differences: *p < 0.05, **p < 0.01, ***p < 0.001.

Following this, the capacity for arborization and progression of iPSC‐SN nerve endings on the matrix surfaces was assessed, aiming at replicating cardiac sympathetic innervation. iPSC‐SNs, labeled with B3‐tubulin, exhibited successful attachment and neurite outgrowth on both matrices (**Figure** **9**A). Moreover, confocal imaging at higher magnification confirmed the biocompatibility and proper sprouting ability of the iPSC‐SN‐generated nerve endings (Figure 9B). Finally, quantitative RT‐PCR experiments revealed no significant differences in MAP2 gene expression levels between iPSC‐SNs encapsulated in the dystrophic matrix and those embedded in the control matrix (Figure 9C). These findings suggest that the dystrophic substrate does not impede the differentiation and maturation of iPSC‐SNs and that the expression of nerve growth factor (NGF) and its high‐affinity (TRKA) and low‐affinity (p75NTR) receptors remains unaltered by the dystrophic substrate.

### 2.5 Multicellular 3D Model to Study the Dystrophic Cardiac Environment

**Figure 10:**
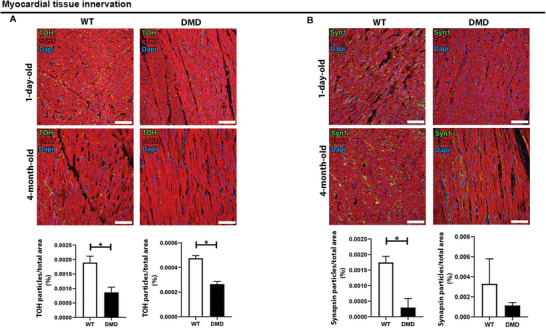
Myocardial tissue innervation. A) Cardiac tissue of WT and DMD pig stained against Synapsin (green) and cardiac troponin T (cTNT; red), or B) TOH (green) and cardiac troponin T (cTnT; red). Nuclei were detected by Dapi (blue). The scale bar represents 50 µm. The graphs indicate the percentage of Synapsin (A) or TOH (B) particles in the total area. Student *t*‐test was used to evaluate the differences between means; *n* = 3 section per *n* = 3 biological replicates; significant differences: *p < 0.05, **p < 0.01, ***p < 0.001.

Histological analysis of the dystrophic porcine tissues employed for DystroGel generation revealed notable disparities in the extension and sprouting capacity of sympathetic terminals that innervate myocardial tissue (**Figure** **10**A,B). Indeed, the analysis revealed that the progression and arborization of nerve endings are significantly reduced (lower percentage of Synapsin and TOH) compared to control. This deficit, previously documented in dystrophic patients, contributes to heart failure and eventual mortality.^[^ ^28^ ^]^

**Figure 11:**
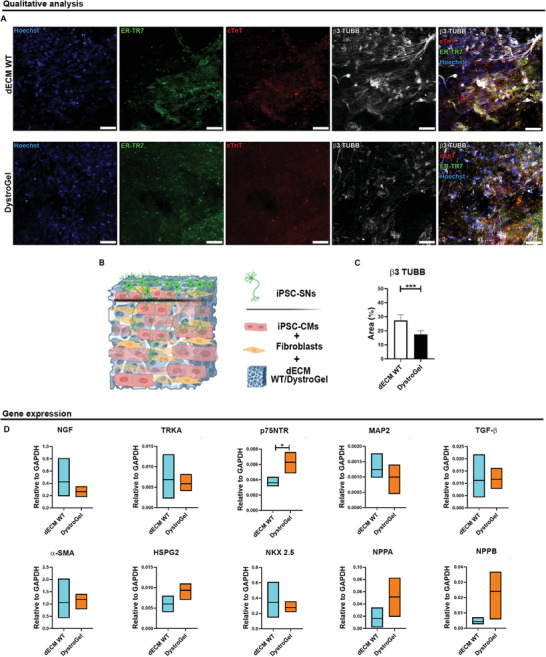
Multicellular 3D Model. A) Z‐stack image reconstruction of the whole WT (upper panel) and DystroGel (lower panel) samples. The scale bars represent 50 µm, respectively. ER‐TR7 (green), cTnT (red), and b3TUBB antibodies were used to stain fibroblasts, iPSC‐CMs, and iPSC‐SNs, respectively. Nuclei were counterstained with Hoechst (blue). B) Representative reconstruction of cellular organization within the 3D triculture model, created using Biorender. C) The graph indicates the area in the percentage of nerve endings labeled with b3TUBB inside dECM WT and DystroGel. Student *t*‐test was used to evaluate the differences between means; *n* = 3 sections per *n* = 3 biological replicates; significant differences: *p < 0.05, **p < 0.01, ***p < 0.001. D) Gene expression analysis related to iPSC‐SNs (NGF, TRKA, p75NTR, and MAP2), fibroblasts (TGF‐b, a‐SMA, and HSPG2) and iPSC‐CMs (NKX 2.5, NPPA and NPPB). Student *t*‐test was used to evaluate the differences between means; *n* = 3; significant differences *p < 0.05, **p < 0.01, ***p < 0.001.

To assess the functional impact of DystroGel for the generation of a neuromuscular junction through a 3D hetero‐cellular approach, we engineered a comprehensive microenvironment, resulting in the creation of a 3D model that replicates the cellular crosstalk of the neurocardiac junction (**Figure** **11**A,B). We co‐resuspended iPSC‐CMs and fibroblasts within DystroGel and control dECM to generate bioinks. Subsequently, iPSC‐SNs were deposited on top of the 3D structures to mimic the nerve endings that reach the heart from the superior root ganglia, establishing connections with myocardial cells. Recently, the cause of cardiac denervation has been linked to the composition of the dystrophic ECM, and replicating this deficit in vitro could advance our understanding and treatment of the disease symptoms.^[^ ^28^ ^]^ The three healthy cell populations were distinguished using specific markers (β3‐tubulin, cTnT, and ER‐TR7) through immunofluorescence assays, and the area occupied by nerve terminals was quantified using ImageJ software (Figure 11C). The analysis revealed that DystroGel significantly reduced the progression and arborization of nerve endings compared to control, affecting the connection between the sympathetic nervous system and myocardium, as observed in the cardiac histology of animal models. These findings are further supported by gene expression data, which indicate an upregulation of p75NTR, a factor known to inhibit neuronal growth (Figure 11D),^[^ ^44^ ^]^ confirming the role of the dystrophic extracellular environment in cardiac denervation. In contrast, a decreasing trend was observed in NGF and its high‐affinity receptor TrkA, supporting the hypothesis that the p75NTR/TrkA ratio is related to cell fate.^[^ ^45^ ^]^ Modifications in NGF expression within the heart contribute to neuronal remodeling, and its production and release are altered under various pathological conditions. NGF also plays a vital role in the crosstalk between the nervous and cardiovascular systems, regulating heart innervation and harmonious contraction. Furthermore, genes associated with remodeling exerted by fibroblasts (TGF‐β, α‐SMA, and HSPG2) show a tendency to return to homeostasis once co‐deposited with CMs. On the other hand, iPSC‐CMs demonstrate a tendency toward stress, as demonstrated by the increased expression of NPPA and NPPB.

## 3 Discussion

3D tissue engineering is a promising approach that enhances our understanding of disease biology and enables comprehensive drug screening through realistic in vitro models. Specifically focusing on the heart, it is crucial to develop microtissues that accurately reflect both physiological and pathological environments. An important aspect of this process is the use of hydrogels or scaffolds derived from the decellularized extracellular matrix (dECM),^[^ ^46^, ^47^ ^]^ which contains essential stromal macromolecules. Notably, the dECM preserves its physicochemical signals and biological properties post‐decellularization, making it an ideal biomaterial for providing mechanical support and a biologically active 3D substrate for cell seeding.^[^ ^47^, ^48^ ^]^ In this work, we used cardiac samples from a recently generated porcine model of DMD^[^ ^26^ ^]^ to create a new pathological dECM‐based hydrogel, called DystroGel. The production and characterization of DystroGel from porcine heart tissues present significant advancements in creating a biocompatible hydrogel tailored for cardiac applications.

Specifically, our refined decellularization protocol effectively eliminated cellular components from both WT and DMD cardiac tissues, as evidenced by the substantial reduction in DNA content (*p* < 0.0001) and the successful visualization of collagen structures through Masson's trichrome and immunofluorescence assays. These results not only confirm the removal of myocytes and nuclei but also demonstrate the preservation of essential ECM components, particularly collagen I and IV, crucial for maintaining the structural integrity and functional properties of the cardiac ECM. The rheological properties of the dystrophic dECM revealed significant differences in viscosity and gelation behavior between the pre‐solutions derived from different ages and genotypes. In particular, the higher viscosity observed in 4‐month‐old pig‐derived dECM compared to 1‐day‐old counterparts suggests age‐related changes in the ECM composition, which could influence the mechanical properties and functionality of the hydrogel. Moreover, the distinct behaviors of DMD samples indicate inherent differences in ECM composition from birth, highlighting the pathological alterations that occur in dystrophic tissues. Importantly, mechanical testing results indicate that Young's modulus of the pathological dECM‐based hydrogel varies significantly with age and genotype, with 4‐month‐old DMD samples exhibiting increased stiffness, reflective of the fibrotic nature of dystrophic cardiac tissues. This increase in stiffness is essential for mimicking the altered biomechanical environment of diseased hearts, providing a more relevant substrate for in vitro modeling.^[^ ^49^ ^]^

Furthermore, proteomic analysis conducted on dECM‐based hydrogels elucidated the complexities of dECM composition in both WT and DMD samples, with over 200 proteins identified across conditions. The strong correlation in total protein profiles among 1‐day and 4‐month‐old WT and DMD samples (mean *r* values of 0.9092 and 0.9293, respectively) underscores the robustness of the proteomic data. Notably, the identified differentially expressed proteins (DEPs) reveal significant dysregulation in pathways related to muscle contraction and cell junction integrity, emphasizing the alterations associated with disease progression and aging. Precisely, the upregulation of proteins such as MYL2, CKM, and MYH7 in DMD 4‐month‐old samples suggests a compensatory response to the pathological state, while the downregulation of crucial proteins in DMD samples reflects impaired contractile function. Moreover, we observed dysregulation of COL5A1 and HSPG2 (perlecan), in the 4‐month‐old DMD samples. The dysregulation of HSPG2, which has been linked to cardiac inflammation, dilated cardiomyopathy, and fibrosis,^[^ ^50^ ^]^ contributes to the reduced contractile capacity of the cardiac muscle and leads to abnormal remodeling of the ECM. This process affects the mechanical properties of cardiac tissue and contributes to heart failure.^[^ ^2^ ^]^ These findings indicate that the 4‐month‐old DMD dECM‐based hydrogel (referred to as DystroGel) successfully replicates the properties of dystrophic cardiac tissue. It displays the heightened stiffness characteristic of fibrotic tissue, along with increased structural integrity that resists disintegration.

The printability tests of DystroGel, using a novel bioprinting platform, demonstrated its potential for creating 3D constructs that mimic the architecture of cardiac tissue. Although this initial test does not fully replicate the complex microenvironment of dystrophic cardiac ECM, it serves as a proof of concept for future applications in tissue engineering. Importantly, although we performed an initial preliminary test, we aim to develop DystroGel further by incorporating specific cell types to provide a more faithful recapitulation of the pathological environment, both from a molecular and architectural perspective. The ability to control the cross‐linking process while maintaining the properties of dECM allows for a versatile approach to fabricating tissue‐engineered constructs, laying the groundwork for more sophisticated models in the future.

In assessing the biocompatibility and functional outcomes of DystroGel in vitro, the encapsulation of fibroblasts, iPSC‐CMs, and iPSC‐SNs within the dystrophic matrix revealed significant insights into cellular behavior in response to the pathological environment. The comparable viability of the different cell types within DystroGel and their ability to maintain critical cardiac markers (e.g., cTnT in iPSC‐CMs) suggest that the hydrogel supports cell survival and functionality. However, the observed upregulation of fibrosis‐associated genes like TGF‐β and COL1A1 in fibroblasts indicates that the mechanical properties of DystroGel may drive pathological remodeling, highlighting the importance of the ECM in influencing cellular responses and behavior.^[^ ^51^, ^52^ ^]^ In addition, the gene expression profile of iPSC‐CMs revealed intriguing upregulation of NKX2.5 and NPPA genes, suggesting that the pathological dECM may play a role in compromising cardiomyocyte maturation and differentiation.^[^ ^15^, ^53^, ^54^ ^]^

Moreover, the establishment of a 3D model that simulates the neurocardiac junction using DystroGel provides a valuable tool for studying cardiac innervation and dysfunction in DMD. The impaired sprouting of iPSC‐SNs on DystroGel compared to dECM WT aligns with the observed cardiac denervation in dystrophic conditions, supporting the hypothesis that ECM composition plays a crucial role in neuronal growth and cardiac function.^[^ ^28^, ^55^ ^]^ The gene expression analysis indicates a shift toward neurotrophic signaling that could inform future therapeutic strategies for addressing cardiac denervation and dysfunction in DMD.^[^ ^35^, ^45^, ^56^ ^]^

Thus, DystroGel represents a significant step toward creating a tailored biomaterial for cardiac tissue engineering, and we believe that the integration of this model into future research efforts will undoubtedly enhance the translational potential of therapeutic approaches aimed at restoring cardiac function in dystrophic conditions.

## 4 Conclusion

In summary, DystroGel represents a pioneering advancement in cardiac tissue engineering, capturing the key pathological features of the ECM specific to DMD. Through its comprehensive mechanical, biochemical, and biological characterization, DystroGel provides a clinically relevant platform that closely simulates the molecular and biomechanical environment of dystrophic cardiac tissue, essential for understanding disease mechanisms and evaluating targeted therapies.

By accurately replicating the diseased ECM, DystroGel bridges the gap between preclinical research and clinical applications. This model enables researchers to assess therapeutic strategies within a context that closely mirrors human pathology, enhancing the predictive value of preclinical studies and providing insights directly applicable to patient care. Its ability to support diverse cell types in a 3D structure further strengthens its utility as a translational research tool, paving the way for more effective and clinically meaningful treatments for DMD and other fibrotic cardiac diseases.

As we continue to refine DystroGel, particularly in printability and cellular integration, its role as a critical model in advancing therapeutic development will expand, offering a path forward in tissue engineering and regenerative medicine with real clinical impact.

## 5 Experimental Section

### Porcine Cardiac Tissue Decellularization

Wild‐type and dystrophic pig heart tissues,^[^ ^26^ ^]^ derived from 1‐day and 4‐month‐old animals, were sampled at the Center for Innovative Medical Models (CiMM), LMU Munich, Germany. All animal experiments were performed according to the German Animal Welfare Act and Directive 2010/63/EU on the protection of animals used for scientific purposes and were approved by the responsible animal welfare authority (Government of Upper Bavaria; permission 55.2‐1‐54‐2532‐163‐2014). For the 1‐day‐old pigs, the entire heart was used, while for 4‐month‐old pigs’ sections from various regions of the heart (right and left ventricles and atria) were used. Cardiac tissue decellularization was conducted following a previously described protocol^[^ ^29^ ^]^ with slight modifications. Briefly, the tissues were cut into pieces ≈1 mm thick. The minced heart tissue was stirred in 0.3% SDS in a Milli‐Q water for 48 h, followed by treatment with a 3% Triton X‐100 (Sigma‐Aldrich, St. Louis, MO, USA; X100‐500ML) solution for a further 48 h. The dECM were washed using Milli‐Q water for at least 3 days, lyophilized, and stored at −80 °C.

### Determination of Decellularization Efficiency—DNA Quantification

Twenty milligrams of each specimen were treated using a GRS Genomic DNA Kit – Tissue kit (Grisp, GK03.0100), according to the manufacturer's instructions, to assess the total DNA content in the dECM compared to native heart samples. DNA samples (*n* = 3) were then quantified using a nanophotometer (ThermoFisher Scientific, Waltham, MA, USA).

### Determination of Decellularization Efficiency—Masson's Trichrome

Masson's Trichrome assay was performed to evaluate the efficacy of the decellularization method. Native porcine heart samples of 1‐day and 4‐month‐old were used as controls. The decellularized fresh tissues were embedded directly in a Tissue Freezing Medium Optimal Cutting Temperature (OCT; Leica Biosystem, 14 020 108 926) and sectioned using a cryostat (Leica Microsystem, Wetzlar, Germania). The sections (6‐µm) were stained with Masson's Trichrome KIT (Sigma‐Aldrich, HT15) according to the manufacturer's protocol.

### Determination of Decellularization Efficiency—Immunofluorescence Assay

Transversal slices of native and decellularized tissues were embedded in OCT, sectioned by a cryostat, and permeabilized in 0.3% TRITON X‐100 (Sigma‐Aldrich) for 10 min at room temperature (RT). The samples were first incubated in 5% Bovine Serum Albumin (BSA) blocking solution for 30 min to saturate the nonspecific sites, and then overnight at 4 °C with anti‐Sarcomeric Alpha Actinin (αSARC; 1:100, Bioss‐Antibodies, bs‐10367R), cardiac Troponin T (cTnT; 1:100, Abcam, ab8295), Collagen I (Col1, 1:100, Novus Biologicals, NB600‐408) and Collagen IV (Col4, 1:100, Abcam, ab6586) antibodies diluted in 0.5% BSA solution. Finally, the sections were exposed to the appropriate secondary conjugated antibodies. Nuclei were detected with Hoechst. A Leica SP5 laser scanning confocal microscope was used to acquire labeled samples.

### dECM‐Based Hydrogel Generation

dECM‐based pre‐hydrogel solutions were generated by mixing 10 mg pepsin (Sigma‐Aldrich, P7125) per 100 mg of lyophilized dECM in 0.5 m acetic acid solution. The pre‐solutions were stirred constantly until the dECM was completely solubilized and formed a homogenous solution, at which point the pH was brought up to 7.4 with 10 m NaOH. The neutralization process was challenging due to the density of the biomaterial and pH standardization. To overcome this problem, dECM solutions were immersed in a bath of isopentane and ice, thus ensuring their liquid state and allowing continuous pH monitoring throughout the neutralization procedure. All the experiments were performed using diluted biomaterials at 1% concentration.

### dECM‐Based Hydrogel Characterization—Rheological Tests

The rheological properties of dECM pre‐solutions derived from WT and DMD pig heart tissues of 1‐day and 4‐month‐old were evaluated by performing shear rate sweep and time sweep tests through a rheometer (Physica MCR 301, Anton Paar). Both tests were performed at 37 °C using a Peltier plate stage and 25 mm parallel plate geometry. About 200 µL of each dECM pre‐solution were deposited onto the stage, and a gap distance of 300 µm was set. Shear rate sweeps were performed in the range of 1–100 s^−1^, and the corresponding viscosity and shear stress values were acquired and plotted as a function of shear rate. The samples were left to equilibrate at the set temperature for at least 5 min before starting the shear rate sweep. The time sweep test was performed imposing a constant frequency of 1 Hz and a constant strain of 5% at 37 °C for 25 min to obtain the storage modulus (G′) akmnd loss modulus (G″) values of the dECM pre‐solutions as a function of time when placed at 37 °C. The test was performed in duplicate for each dECM pre‐solution for both shear rate and time sweeps.

### dECM‐Based Hydrogel Characterization—Compression Tests

The stiffness of the dECM derived from WT and DMD pig's heart tissues of 1‐day and 4‐month‐old was evaluated by performing uniaxial compression tests carried out on the dECM‐derived samples previously hydrated in PBS at RT. The tests were performed using a universal testing machine (ZwickiLine 1 kN, Zwick Roell) equipped with a 10N load cell in displacement control up to 75% deformation, with a displacement velocity of 2 mm min^−1^. The Young's modulus of the different dECM matrices was calculated for each sample as the slope of the initial linear part of the stress–strain curve (0–5%). The test was performed in triplicate for each matrix.

### dECM‐Based Hydrogel Characterization—In Vitro Stability

Nonenzymatic degradation of the hydrogel over time was evaluated by stability test. The bulk samples were hydrated in PBS and incubated at 37 °C, monitoring the weight over 30 days. The percentage of weight loss (WL) was calculated according to the following formula:

(1) $$\mathrm{WL}\left(\%\right)=\left[\left(W0-\mathrm{Wi}\right)/W0\right]\times 100$$

where W0 is the initial weight of the sample after thermal gelation (*t *= 0) and Wi is the weight of the hydrogel at different time points.

### dECM‐Based Hydrogel Characterization—Morphological Analysis and Porosity Measurement

For the microstructural characterization, the samples were dehydrated using graded ethanol concentrations of 20%, 50%, 70%, 90%, and 100% for 5 min each. The ethanol was then replaced with liquid carbon dioxide and sublimed entirely using critical point drying (K850 CPD, Quorum Technologies). A cross‐section of ≈15 mm was cut in half with a razor, adhered to double‐sided carbon tape on aluminum stubs, and sputter‐coated with 10 nm gold (CCU‐010 LV, Safematic) to prevent charge accumulation. The sample's morphology was then examined using a high‐resolution Zeiss Sigma 300 VP field‐emission scanning electron microscope (FE‐SEM, Carl Zeiss AG, Oberkochen, Germany) in secondary electron (SE) mode.

ImageJ software was employed for pore diameter analysis. The mean and standard deviation of the Feret diameters of the pore sizes in the WT and DMD samples were calculated by applying threshold filters and particle analyzing functions on the SEM images.^[^ ^57^ ^]^

### dECM‐Based Hydrogel Characterization—3D Wet‐Spinning Bioprinting

Core/shell hydrogel fibers were fabricated, and 3D spatially deposited using a custom 3D wet‐spinning bioprinter.^[^ ^34^ ^]^ Briefly, the platform consisted of several components, including a 3D‐printed MPH with a crosslinking bath microtank. The MPH was equipped with a co‐axial nozzle located at its base, which facilitated the immediate gelation of extruded core/shell fibers. Both the inner and outer needle diameters were 500 µm. The samples were collected on a rotating drum collector with dimensions of 20 mm in diameter and 180 mm in length. The platform also included an *X*‐axis with a travel range of 160 mm. Arduino Mega board and custom Python software were used to control the entire system. The core bioink was composed of 1% dECM while the shell biomaterial ink was prepared by dissolving 2% w/v low molecular weight alginate (LMW‐ALG), 0.5% w/v high molecular weight alginate (HMW‐ALG) in 25 mm HEPES. Prior to use, the MPH and tubing were first washed with 70% ethanol solution and sterile water. The inks were extruded at constant flow rates (Qcore = 160 µL min^−1^, Qshell = 320 µL min^−1^) to generate the co‐flow in the extrusion nozzle. The crosslinking bath microtank was filled with 0.6 m Calcium chloride (CaCl_2_) solution favoring the instantaneous gelation of the core/shell fibers in the proximity of the tip of the nozzle. The rotation speed of the drum was set to 64 rpm and each sample was composed of 40 threads. To crosslink the dECM in the core, the bundles were incubated for 30 min at 37 °C in a 25 mm HEPES buffer solution.

### Mass Spectrometry Analysis—Sample preparation

Samples were prepared to nanoscale liquid chromatography coupled to tandem mass spectrometry (nLC‐MS/MS) analysis with the MS‐compatible detergent RapiGest SF Surfactant (Waters Corporation, Milford, MA, USA; 186 001 861). Briefly, after recovering samples from the −80 °C freezer, they were centrifuged at 13 rpm, for 10 min and the supernatant was discarded. Pellets were resuspended in 0.1 m Ammonium bicarbonate (NH_4_HCO_3_), pH = 7.9 and mechanically homogenized. RapiGest SF was added to each sample to reach a final concentration of 0.2% v/v and samples were heated at 95 °C for 20 min. After centrifugation at 13 rpm for 10 min, samples were quantified using Qubit Protein Assay Kit on a QubitTM4 Fluorometer (Invitrogen, ThermoFisher Scientific, Waltham, MA, USA); 50 µg of proteins were recovered from each sample and digested overnight with trypsin (Trypsin Gold, Promega, Madison, WI, USA; V5280) 1:50 enzyme/substrate ratio. Tryptic digestion was stopped with trifluoroacetic acid (TFA) to reach a final concentration of 0.5% v/v and samples were centrifuged (13 rpm for 10 min) to remove any matrix debris. Eventually, resulting peptides were desalted and enriched with PepClean C18 Spin Columns (ThermoFisher Scientific, Waltham, MA, USA), according to the manufacturer's instructions.

### Mass Spectrometry Analysis—nLC‐MS/MS Analysis

Each sample was analyzed in two technical replicates on LC‐MS/MS platform: Eksigent nanoLC‐Ultra 2D System (Eksigent, Dublin, CA, USA) for nano liquid chromatography coupled with LTQ Orbitrap XLTM (Thermo Fisher Scientific, San Jose, CA, USA) for MS/MS analyses. In particular, peptides were separated with the following eluent gradient: (A) 0.1% formic acid in water; (B) 0.1% formic acid in acetonitrile; the gradient profile was 10–50% B in 104 min, 50–95% B in 17 min; 95% B for 9 min. Peptides were analyzed by tandem MS following the previously reported methods.^[^ ^58^ ^]^

### Mass Spectrometry Analysis—Data Processing

The experimental tandem mass spectra (MS/MS) produced by LC‐MS/MS analysis were matched against the in‐silico tryptic peptide sequences of the Sus scrofa protein database (46179 entries) retrieved from UNIPROT (www.uniprot.org) in December 2022. Data processing was performed by Proteome Discoverer 2.1 software (Thermo Fisher Scientific), based on the SEQUEST algorithm.^[^ ^59^ ^]^ Matches between spectra were only retained if they had a minimum Xcorr of 2 for +1, 2.5 for +2, and 3.5 for +3 charge state, respectively. Percolator node was used with a target–decoy strategy to give a final false discovery rate at a peptide spectrum match level of 0.01 (strict) based on *q*‐values, considering a maximum deltaCN of 0.05.^[^ ^60^ ^]^ Only peptides with a minimum peptide length of six amino acids and Rank 1 were considered, while peptide confidence was set to “medium”. Protein grouping and strict parsimony principle were applied. Results were then exported to an Excel file for further processing.

### Mass Spectrometry Analysis—Label‐Free Quantitation and PPI Network Analysis

The spectral count (SpC) values of the identified proteins were normalized using a total signal normalization method and compared using a label‐free quantification approach, as previously reported.^[^ ^61^, ^62^ ^]^ In detail, the considered protein lists (WT 1D, *n* = 4; WT 4M, *n* = 4, DMD 1D, *n *= 4; DMD 4M, *n* = 4,) were first processed by linear discriminant analysis (LDA) and proteins with the largest (≥6) F ratio and smallest *P*‐value (≤0.05) were retained and considered differentially expressed with high confidence. In addition, pairwise comparisons (DMD 1D versus WT 1D, DMD 4M versus WT 4M, WT 4M versus WT 1D, DMD 4M versus DMD 1D, WT 4M versus DMD 1D, and DMD 4M versus WT 1D) were evaluated by Dave and DCI indices. Proteins selected by LDA were processed by hierarchical clustering applying Ward's method and a Euclidean distance metric using JMP15.2 software. Finally, a PPI network was built by combining differentially expressed proteins and the Sus scrofa PPI network was retrieved and functionally analyzed by STRING Cytoscape's app;^[^ ^60^ ^]^ only experimentally and database‐defined PPI with a score >0.15 were considered, while GO process, KEGG, Reactome and Wikipathways results were investigated for identifying functional modules.

### In Vitro Experiments—Human Fibroblast Culture

Human fibroblasts were cultured in Dulbecco's Modified Eagle Medium (DMEM; Gibco; 11 965 092), 10% fetal bovine serum (FBS, Gibco; 10 270 106) and 1% Penicillin‐Streptomycin (5000 U mL^−1^; Corning; 30‐002‐CI) and expanded to obtain a sufficient number to perform the experiments.

### In Vitro Experiments—iPSC Culture

UndifferentiatediPSC available in the laboratory were cultivated and expanded on plates pre‐coated with 10 µg mL^−1^ Vitronectin XF (STEMCELL Technologies, 0 7180) diluted in cell adhere dilution buffer (STEMCELL Technologies). Cells were maintained in TeSR‐E8 medium (STEMCELL Technologies, 0 5990) and cultured at 37 °C, 5% CO_2_. The medium was refreshed daily, and iPSCs were passaged at 80% confluence by treatment with EDTA.^[^ ^23^ ^]^

### In Vitro Experiments—iPSC Differentiation toward CM Phenotype

The Cardiomyocyte Differentiation Kit (STEMCELL Technologies, 0 5010) was employed for the differentiation of iPSCs into CM. Briefly, iPSCs were dissociated with Gentle Cell Dissociation Reagent (STEMCELL Technologies) at 37 °C for 8 min. A single‐cell suspension was obtained, pipetting up and down 3–4 times using a pipette with a 1000 µL tip. Then, the cells were washed in TeSR‐E8 complete medium and centrifuged at 300 × g for 5 min. The iPSCs were resuspended in a fresh Tesr‐E8 medium supplemented with RevitaCell (GIBCO, A2644501), plated 8 × 10^5^ cells well^−1^ on pre‐coated Matrigel (Corning) 12‐well plates and incubated at 37 °C. After 24 h, the medium was replaced with fresh TeSR‐E8 complete medium without RevitaCell. Following a further 24 h, iPSCs were sequentially exposed to the Differentiation Medium A (48 h), Differentiation Medium B (48 h), and Differentiation Medium C (48 + 48 h). From Day 10, the medium was changed to Maintenance Medium and was refreshed every other day.

### In Vitro Experiments—iPSC Differentiation toward SMs Phenotype

SNs were differentiated from iPSC according to previously published protocols.^[^ ^63^ ^]^ Briefly, cells were plated on Geltrex‐coated 12‐well plates and cultured in a complete mTeSR1 medium until they reached 70–80% confluency. On day 0 (starting of induction), the medium was switched to StemFlex, and cells were cultured for 2 weeks adding sequentially different inhibitors and small molecules. Starting from day 4 to day 12, the medium was gradually changed (25% increments every other day) toward N2‐supplement‐enriched B‐27 plus medium. On day 12, the cells were detached and transferred to Geltrex‐coated 24‐well plates in a neuronal medium consisting of B‐27 plus neuronal medium supplemented with 2 mm *L*‐glutamine, N‐2 supplements, 0.2 mm ascorbic acid, 0.2 nm dbcAMP, 10 ng mL^−1^ NGF, 10 ng mL^−1^ BDNF, and 10 ng mL^−1^ GDNF. Finally, BMP4 was added for the first two days. The cells were maintained in a neuronal medium without a BMP4 supplement from day 14 until the end of neuronal maturation (Day 40).

### In Vitro Experiments—3D Culture

dECM WT and DystroGel were used as supporting biomaterials to generate 3D bulk samples. Fibroblasts and iPSC‐derived CMs were resuspended in each sterile bioink at a concentration of 20 × 10^6^ and 30 × 10^6 ^cells mL^−1^, respectively, poured in Polydimethylsiloxane (PDMS) molds of 200 µL, and polymerized at 37 °C for 30 min. Conversely, 2 × 10^5^ iPSC‐SNs were seeded on the surface of the 3D bulks. Finally, the samples were cultivated for 15 days and used to perform viability tests, immunofluorescence, and gene expression assays. Fibroblasts, iPSC‐derived CMs, and iPSC‐SNs cultured in standard conditions (2D) were used as controls. The triculture constructs were prepared by mixing fibroblasts with iPSC‐derived CMs in either WT or DMD bioink at a 10 × 10^6^ and 30 × 10^6 ^cells mL^−1^ concentration, respectively. This mixture was then poured into PDMS molds of 200 µL each and polymerized at 37 °C for 30 min. After 1 week of culture, 2 × 10^5^ iPSC‐SNs were seeded onto the surface of these 3D constructs. The triculture samples were cultured in a co‐culture medium consisting of three parts: Neuronal, iPSC CMs, and fibroblasts medium supplemented with 10 ng ml^−1^ NGF (SRP3018, Sigma‐Aldrich) for a further 7 days encouraging the innervation process.

### Live/Dead Assay

The viability of the cell encapsulated in the biomaterials was evaluated by LIVE/DEAD Viability/Cytotoxicity Kit (Invitrogen, L3224) after 15 days of culture. Two microliters of ethidium homodimer and 0.5 µL of calcein AM were diluted in 998 µL PBS, then 300 µL were administered to bulks and incubated for 45 min in 5% CO_2_ at 37 °C. Living and dead cells were observed by Leica SP5 laser scanning confocal microscope (Leica Microsystem). Living cells were detected by calcein AM (green), and death cells by EthD‐1 (red). The number of viable cells was quantified using ImageJ software. The percentage of viability was determined by dividing the number of viable cells by their total count within the area.

### Immunofluorescence Assay

The bulk constructs were fixed in 4% Paraformaldehyde (PFA) for 1.3 h and incubated in 5% Bovine Serum Albumin (BSA) blocking and 0,2% TRITON X‐100 solution for 2 h at RT. Then, the samples were incubated overnight at 4 °C with the following primary antibodies diluted 1:100: Vimentin (Sigma‐Aldrich, V5255); cardiac Troponin T (cTnT, Abcam, ab8295); β3‐Tubulin (β3TUBB, Biologend, 802 001), Fibroblasts marker (ER‐TR7, Santa Cruz Biotechnology, sc‐73355) Thereafter, the bulks were exposed to appropriate secondary antibodies (ThermoFisher, Waltham, MA, USA) for 2 h at RT. Nuclei were counterstained with Hoechst (1:500) for 1 h.

### Image Acquisition and Processing

The acquisition of entire 3D samples was performed utilizing a high‐resolution custom‐assembled spinning disk confocal microscope. This system comprised a Nikon Ti inverted microscope equipped with a Crest Optics X‐light V2/VCS scan‐head, which was coupled to an Andor DU888 EMCCD camera, configured with a 70‐micron pinhole, and to LDI light source (89‐North LDI). The imaging process was conducted at 10× magnification, capturing Z‐stacks with a 10‐micron Z‐step size to encompass the complete volume and area of the samples. Subsequent processing and analysis of the acquired datasets were performed using NIS‐Elements v.5.30 software (Nikon‐Lim). Specifically, the images underwent the NIS‐Elements 2D Automatic Deconvolution Algorithm. Furthermore, a maximum intensity projection was employed for whole sample images, followed by the conversion of the multi‐point file into a single large image utilizing the XY coordinates. Ultimately, NIS‐Elements v.5.30 software was also employed to execute the 3D volume rendering process.

A Leica SP5 laser scanning confocal microscope was also used to acquire labeled 3D samples. The 3D bulks were acquired at 20× magnification + 2× zoom. Neuronal growth assessment analyses were performed using ImageJ software relating the area covered by SNs to the field total area.

### Gene Expression Analysis

Total RNA was extracted from the cells of each experimental condition (*n* = 3) using RNeasy Plus Universal Mini Kit (Qiagen) according to the manufacturer's protocol. RNA concentration was determined using a NanoDrop UV–vis spectrophotometer. A 1 µg quantity of total RNA was reverse transcribed to cDNA using the High‐Capacity cDNA Reverse Transcription Kit (Applied Biosystem). The evaluation of gene expression was performed by quantitative RT‐PCR using QuantStudio 1 Real‐Time PCR System (ThermoFisher Scientific). All reactions were carried out in 10 µl reaction volume and in duplicate. The expression data were normalized using the Ct values of GAPDH as the housekeeping gene. The primer sequeces are listed in **Table** **1**.

**Table 1:** Human primer sequences for quantitative RT‐PCR analysis.

Gene symbol	Sense‐forward primer	Antisense‐reverse primer
GAPDH	TCTTTTGCGTCGCCAGCCGAC	TGACCAGGCGCCCAATACGAC
FN	AGAGTGGAAGTGTGAGAGGC	GGTAAACAGCTGCACGAACA
TGFβ	CAGCAGGGATAACACACTGC	CATGAGAAGCAGGAAAGGCC
COL1A1	CCCCAGCCACAAAGAGTCTA	TACCTGAGGCCGTTCTGTAC
αSMA	CTGCTGAGCGTGAGATTGTC	TCAAGGGAGGATGAGGATGC
NKX2.5	TTAAGTCACCGTCTGTCTCCCTCA	ACCGACACGTCTCACTCAGCATTT
NPPA	ACAATGCCGTGTCCAACGCAGA	CTTCATTCGGCTCACTGAGCAC
NPPB	TCTGGCTGCTTTGGGAGGAAG	CCTTGTGGAATCAGAAGCAGGTG
cTnI	TTCGAGGCAAGTTTAAGCGG	TTTCCTTCTCGGTGTCCTCC
MAP2	CAAATGTGGCTCTCTGAAGAACA	GGGCCTTTTCTTTGAAATCTAGTTT
NGF	TGTGGGTTGGGCATAAGACC	CTCTCCCAACACCATCACCT
TRKA	TTCCATTTCACTCCTCGGCT	CCAGAGCGTTGAAGGAGAGA
P75NTR	CCGACAACCTCATCCCTGT	TCCTTGCTTGTTCTGCTTGC

### Statistical Analysis

A statistically significant number of repetitions were performed for each measurement, usually three unless otherwise stated. Data were presented as mean ± standard error mean (SEM). The statistical significance was assessed by the student's *t*‐test (for two experimental groups) and by the repeated measure ANOVA with Tukey correction (differences among more groups). Statistical analysis was accomplished using Prism 8 (GraphPad Software, La Jolla, CA, USA). Differences were displayed as statistically significant when *p*‐values (*p*) < 0.05. Statistically significant values were presented as *p < 0.05, **p < 0.01, ***p < 0.001, and ****p < 0.0001.

## Conflict of Interest

The authors declare no conflict of interest.

## Author Contributions

R.R. conceptualized the study and designed the experiments. R.R. and C.B. acquired funding for the project and supervised all generation, collection, and analyses of research data. M.C. performed all the experiments, analyzed the data, and prepared the figures and tables. R.R., C.B., and M.C. wrote and edited the manuscript. F.M., M.G.C., M.M., S.B., and N.F. conducted the in vitro experiments and supported data analysis. M.S. and E.W. provided the wild‐type and dystrophic pig heart tissues. A.F. provided imaging support. M.C., F.S., F.G., and M.V. performed the compression, stability, and rheological tests. R.V., F.B., P.M., and D.D.F. performed mass spectroscopy and proteomic data analysis. E.D.F. provided experimental support.

## Data Availability

The data that support the findings of this study are available from the corresponding author upon reasonable request.
